# Poster Session II - A239 IMMIGRANT STATUS AND WORK PRODUCTIVITY LOSS IN PATIENTS WITH INFLAMMATORY BOWEL DISEASE: A RETROSPECTIVE COHORT STUDY

**DOI:** 10.1093/jcag/gwaf042.239

**Published:** 2026-02-13

**Authors:** D T Sheka, S Bharatselvam, A N Sasson, M Cino, P Tandon

**Affiliations:** Medicine, University of Toronto, Toronto, ON, Canada; Toronto Western Hospital, Toronto, ON, Canada; Toronto Western Hospital, Toronto, ON, Canada; Toronto Western Hospital, Toronto, ON, Canada; Toronto Western Hospital, Toronto, ON, Canada

## Abstract

**Background:**

Inflammatory bowel disease (IBD) arises during peak working years and can impair occupational productivity. Immigrants may face unique barriers that compound this burden, but the relationship between immigration status and work impairment is poorly understood in IBD.

**Aims:**

To evaluate differences in work productivity between immigrants and non-immigrants with IBD, and to explore whether disease features, psychosocial factors, or immigration-related variables explain observed disparities.

**Methods:**

We conducted a retrospective cohort study of adult patients with ulcerative colitis (UC) or Crohn’s disease (CD) who completed the Work Productivity and Activity Impairment (WPAI) questionnaire during a prospective registry initiative. Baseline characteristics included disease phenotype (described by the Montreal classification), biochemical parameters (such as C-reactive protein, fecal calprotectin), and psychosocial indices (GAD-7 (General Anxiety Disorder-7), PHQ-9 (Patient Health Questionnaire-9), PSS (Perceived Stress Scale), FACIT-F (Functional Assessment of Chronic Illness Therapy-Fatigue Scale)). The primary exposure was immigration status (reported by participants). We explored the primary outcome of composite work productivity loss (absenteeism and presenteeism). We also assessed individual components of the WPAI questionnaire such as where participants immigrated from (Western vs. Non-Western countries). Statistical analyses included Mann–Whitney U to compare continuous variables and chi-square tests for categorical variables. A two-sided p value < 0.05 was considered statistically significant.

**Results:**

163 patients were included of which 50 (31%, mean age 48.7 ± 17.6 years) were immigrants and 113 (69%, mean age 48.1 ± 15.4 years) were non-immigrants. There were no differences in CRP, fecal calprotectin, and psychosocial scores between the two groups. Compared to non-immigrants, immigrant patients with IBD had higher composite productivity loss compared with non-immigrants (87.6% vs. 74.4%, p = 0.032). Compared to those immigrating from Western countries, those immigrating from Non-Western countries were more likely to report absenteeism (16.7% vs. 2.9%), presenteeism (92.5% vs. 83.3%), and composite productivity loss (90.5% vs. 75.8%). Among UC patients, immigrants had more left-sided (E2 = 34.6%) and extensive colitis (E3 = 42.3%) compared with non-immigrants (E2 = 23.4%, E3 = 36.2%), while non-immigrants more often had proctitis (E1 = 40.4% vs 23.1%).

**Conclusions:**

Immigrant patients with IBD experience greater overall productivity loss despite similar disease activity and psychosocial profiles. These findings suggest immigrant status may function as a social determinant of work-related disability, highlighting the need for equity-focused interventions in clinical and workplace settings.

**Funding Agencies:**

None

